# Is Polymorphism in the Apoptosis and Inflammatory Pathway Genes Associated With a Primary Response to Anti-TNF Therapy in Crohn’s Disease Patients?

**DOI:** 10.3389/fphar.2020.01207

**Published:** 2020-08-14

**Authors:** Michal Walczak, Liliana Lykowska-Szuber, Marianna Plucinska, Kamila Stawczyk-Eder, Oliwia Zakerska-Banaszak, Piotr Eder, Iwona Krela-Kazmierczak, Michal Michalak, Marek Zywicki, Wojciech M. Karlowski, Marlena Szalata, Agnieszka Dobrowolska, Ryszard Slomski, Marzena Skrzypczak-Zielinska

**Affiliations:** ^1^Institute of Human Genetics, Polish Academy of Sciences, Poznan, Poland; ^2^Department of Gastroenterology, Dietetics and Internal Medicine, Poznan University of Medical Sciences, Poznan, Poland; ^3^Department of Computational Biology, Faculty of Biology, Institute of Molecular Biology and Biotechnology, Adam Mickiewicz University, Poznan, Poland; ^4^Department of Computer Sciences and Statistics, Poznan University of Medical Sciences, Poznan, Poland; ^5^Department of Biochemistry and Biotechnology, University of Life Sciences, Poznan, Poland

**Keywords:** Crohn’s disease, biological therapy, genetics, response, anti-TNF drugs, next-generation sequencing, apoptosis

## Abstract

Anti-tumor necrosis factor (TNF) therapy is used for the induction and maintenance of remission in Crohn’s disease (CD) patients. However, primary nonresponders to initial treatment constitute 20%–40% of cases. The causes of this phenomenon are still unknown. In this study, we aimed to determine the genetic predictors of the variable reactions of CD patients to anti-TNF therapy. Using long-range PCR libraries and the next-generation sequencing (NGS) method, we performed broad pharmacogenetic studies including a panel of 23 genes (*TNFRSF1A*, *TNFRSF1B*, *CASP9*, *FCGR3A*, *LTA*, *TNF*, *FAS*, *ADAM17*, *IL17A*, *IL6*, *MMP1*, *MMP3*, *S100A8*, *S100A9*, *S100A12*, *TLR2*, *TLR4*, *TLR9*, *CD14*, *IL23R*, *IL23*, *IL1R*, and *IL1B*) in a group of 107 diagnosed and clinically characterized CD patients following anti-TNF therapy. In the studied group, we indicated, in total, 598 single nucleotide variants for all analyzed genomic targets. Twelve patients (11.2%) did not respond to the induction therapy, which was associated with alleles in 11 *loci* located in *FCGR3A* (rs7539036, rs6672453, rs373184583, and rs12128686), *IL1R* (rs2041747), *TNFRSF1B* (rs5746053), *IL1B* (rs1071676, rs1143639, rs1143637, and rs1143634), and *FAS* (rs7896789) genes. After multiple comparison corrections, the results were not statistically significant, however for nonresponders the alleles distribution for those *loci* presented large differences and specified scheme compared to responders and populations. These findings require further investigation in an independent larger cohort before introducing them for a clinical setting, however, we identified an interesting direction. Polymorphism of the *FCGR3A*, *IL1R*, *TNFRSF1B*, *IL1B*, and *FAS* genes could be a predictor of the primary response to anti-TNF therapy in CD patients.

## Introduction

The use of anti-tumor necrosis factor (anti-TNF) agents has revolutionized the treatment of inflammatory bowel diseases (IBDs), which include Crohn’s disease (CD). These drugs induce clinical and mucosal remission, as well as modify the natural course of the disease. Anti-TNF agents are used in patients who present high disease activity and are intolerant of or do not respond to corticosteroids and/or immunosuppressants (Itoh et al., 2001; Hanauer et al., 2002; Colombel et al., 2007).

Not all patients benefit from anti-TNF treatment. Registration results show that about 20%–40% of patients with CD do not respond to the initial therapy, whereas another 40% develop a secondary loss of response with ongoing treatment (Travis et al., 2006; Katsanos et al., 2019). The mechanism of primary nonresponse remains unknown. Currently, much attention has been paid to the pharmacokinetics of biological drugs, which undoubtedly play a role in the long-term response. However, it seems that other mechanisms are involved in the induction phase, including genetic conditions, whereas drug pharmacokinetics is a secondary phenomenon (Drabik et al., 2016; Yamamoto-Furusho, 2017; Linares-Pineda et al., 2018). The etiopathogenesis of CD is not fully understood. However, the regulation of the apoptosis of intestinal inflammatory cells and the regulation of selected inflammation are of vital importance (Itoh et al., 2001; Atreya et al., 2011).

Infliximab and adalimumab are monoclonal antibodies that bind to both the soluble and transmembrane form of TNF (Billmeier et al., 2016). The function of these drugs is not only to neutralize the TNF cytokine, but also to affect a number of other processes in inflammatory cells. They influence the regulation of the disturbed apoptosis process and the decrease in the concentration of proinflammatory cytokines other than TNF (e.g., IL-6, IL1β, and IL-12) (Van Deventer, 2001; Mitoma et al., 2005; Schmitt et al., 2019). On the basis of numerous studies, it has been shown that various proinflammatory cells do not respond properly to the signal of apoptosis and, as a result, they are not eliminated. This defect primarily affects lymphocytes and monocytes (Ferreira et al., 2010; Pott and Maloy, 2018). The apoptotic signal is transmitted by two ways: (1) the external, which is dependent on the receptors, and (2) the internal, which is dependent on cytochrome c. The receptors that are involved in the response to apoptosis include Fas, TNF, and IL-1R. The apoptotic signal is transmitted to the effector enzymes called caspases. Infliximab, through the up-to-now unknown mechanism, activates both external (procaspase-8) and internal (procaspase-9) routes. Consequently, the activation of the central effector caspases (caspase-3) and cell apoptosis occurs (Hengartner, 2000; Wittkopf et al., 2013; Ruder et al., 2019). In addition, enzymes belonging to the group of metalloproteinases are also responsible for the degradation and remodeling of the extracellular matrix. Disorder of their function may lead to prolonged inflammatory infiltration. Furthermore, the substrate specificity of metalloproteinases is also not known. However, it has been established that in terms of apoptosis and anti-TNF drugs, they can alter the membrane-bound death ligands’ activity through the degradation of the extracellular matrix. In fact, anti-TNF therapy has been shown to decrease the activity of metalloproteinases (Black et al., 1997).

In the future, demonstrating the relationship between the genetic variants and the reaction type to anti-TNF treatment may allow for a better personalization of therapy and the avoidance of qualification for treatment of patients who will not respond to a given type of biological therapy. In this paper, in order to practically indicate the pharmacogenetic biomarker of anti-TNF therapy, an attempt was made to determine the influence of the panel gene variability (*TNFRSF1A*, *TNFRSF1B*, *CASP9*, *FCGR3A*, *LTA*, *TNF*, *FAS*, *ADAM17*, *IL17A*, *IL6*, *MMP1*, *MMP3*, *S100A8*, *S100A9*, *S100A12*, *TLR2*, *TLR4*, *TLR9*, *CD14*, *IL23R*, *IL23*, *IL1R*, and *IL1B*) involved in the processes described above in response to this particular biological therapy in CD patients.

## Material and Methods

### Patients and Samples

One hundred and seven Polish patients hospitalized at the Department of Gastroenterology, Dietetics, and Internal Medicine, Poznan University of Medical Sciences in Poznan, Poland, were enrolled in this study. All individuals gave their written consent to genetic testing and the evaluation of biochemical parameters in serum and colonoscopy examination. The research was approved by the Bioethical Committee of the University of Medical Sciences in Poznan, Poland, under Resolution No. 762/13.

We studied patients with a confirmed diagnosis of CD. Diagnosis of the disease was based on medical history, physical examination, endoscopic and MR enterography. The subjects were treated with anti-TNF for the therapeutic program of the National Health Fund (which is an official reimbursement program for all biological therapies in Poland) at the Department of Gastroenterology between 2013 and 2017. The inclusion criteria for patients were that they had to be aged >18 years, have a diagnosis of active CD, be biologic-naïve, as well as having had treatment failure or intolerance to first-line therapies, such as mesalamine, corticosteroids, and/or immunosuppressants. The exclusion criteria were the presence of an ileostomy or colostomy and infectious complications (including intraabdominal infections). The diagnosis was based on previously defined criteria (Gomollón et al., 2017). Clinical disease activity was assessed by using the Crohn’s Disease Activity Index (CDAI) (Best et al., 1976). Patients who had never smoked or quit smoking at least 10 years prior to participating in the study were considered nonsmokers. The patients were administered infusions of infliximab (IFX) at a dose of 5 mg/kg body weight at Weeks 0, 2, 6 (the induction phase), and then every 8 weeks until one year (54 weeks—the maintenance phase). Adalimumab (ADA) was given subcutaneously at Week 0 at a dose of 160 mg, 80 mg was given at Week 2, and then every other week a 40 mg per dose was given until one year (54 weeks).

The anti-TNF treatment response was assessed following 12 weeks of the therapy. The CDAI score was used to determine clinical response. The clinical response was defined as CDAI reduction by ≥70 points. In patients with fistulas, a complete response was defined as a complete cessation of all fistula drainage, whereas a partial response was identified as a decrease of at least 50% but not draining all fistulas.

We also assessed the biological parameter (C Reactive Protein, CRP), endoscopy (Simple Endoscopic Score for Crohn’s Disease, SES-CD), and MRI (Simple Enterographic Activity Score for Crohn’s Disease, SEAS-CD) response (Daperno et al., 2004; Eder et al., 2013). We evaluated these parameters twice—before treatment and after 12 weeks of the induction therapy.

Genomic DNA from all subjects was isolated from peripheral blood, according to standard procedures, using the method with guanidine isothiocyanate and stored at 4°C in a Tris-EDTA (TE) buffer containing 1.0 mM EDTA and 10 mM Tris-Cl until use.

### Long-Range (LR) PCR Amplification

The amplification of the *TNFRSF1A*, *TNFRSF1B*, *ADAM17*, *CASP9*, *FCGR3A*, *LTA*, *TNF*, *FAS*, *IL1B*, *IL17A*, *IL6*, *MMP1*, *MMP3*, *S100A8*, *S100A9*, and *S100A12* genes was performed in 23 fragments using previously described primers (Walczak et al., 2019). Additionally, the primers for *TLR2*, *TLR4*, *TLR9*, *CD14*, *IL23*, *IL23R*, and *IL1R* genes containing exons, splice junctions, and promoters, as well as 5’ and 3’ flanking sequences, were designed (**Table 1**). The LR-PCR conditions were established, and they are presented in **Table 2**.

**Table 1:** Characteristics of amplicons and primer sequences of *TLR2*, *TLR4*, *TLR9*, *CD14*, *IL23*, *IL23R*, and *IL1R* genes.

Frag. No.	Gene	OMIM No.	Chromosome coordinates (GRCh37/hg19)	Primer sequence (5′→3′)	Amplicon length (bp)
1.	*TLR2*	603028	chr4:154620045+154627075	F:CTGGTCTTTCTATAGGGTTAGGCTTT	7031
				R:TCTCTTAGCAGGAAGAAAGAATGACA	
2.	*TLR4*	603030	chr9:120466961+120479323	F:ACTCAAGAAGCCACAGAGATCAAATA	12363
				R:ACTAGTACATGAGACATGGAAACACA	
3.	*TLR9*	605474	chr3:52254989-52260328	F:CTAGATCAGAAGGAGAGTGGGAAGAA	5340
				R:CTGTCCCGTCTTTATGGCAATTTTC	
4.	*CD14*	186940	chr5:140011176-140015540	F:AAAGTTCCCAGCCAAAAGCTATATTC	4365
				R:TTCTTTTCTTGAGGAGGACAGATAGG	
5.	*IL23R (part 1)*	607562	chr1:67627253+67635706	F:CAAACTACGTACTCCCTTGCATATTT	8454
				R:AGAGCAACATTTTAAATCTAACGGGG	
6.	*IL23R (part 2)*		chr1:67647462+67649273	F:CTTTGATGATGGTGATGTACAGATGG	1812
				R:AGAAGAAGAATAGAGCAGGGATTTAAGCA	
7.	*IL23R (part 3)*		chr1:67665995+67673339	F:GATTACAAGCGTGAGCCATAAATTCT	7345
				R:CAAAAGGCTCTTCTTACTTAGTGGTG	
8.	*IL23R (part 4)*		chr1:67684715+67685915	F:CACATTTGGACTTTCTGGAGATTTGA	1201
				R:CATGCCCAGCTAATTTTCGTATTTTT	
9.	*IL23R (part 5)*		chr1:67702124+67706145	F:CATGAAATTAGTGGACACACCTTGA	4022
				R:GAAAGACATTTGTAGAGAGTTTGGCA	
10.	*IL23R (part 6)*		chr1:67720545+67724949	F:GGGAAAAAGTGGGATGTTTATGACTT	4405
				R:TGAAAACATGTGTCCATGTGAAGATT	
11.	*IL23*	605580	chr12:56728218+56734024	F:AAACTCGCTGGCATTTCCTATTATTT	5807
				R:TAATTTTCAACATATGCAGGTCCCAC	
12.	*IL1R*	147810	chr2:102770366+102783183	F:ATTCACACTTCACTCATGTGTTCTTC	12818
				R:AATGAACAGGAGGTAGATTCTGGAAA	

F, forward; R, reverse; chr, chromosome; OMIM, Online Mendelian Inheritance in Man; frag., fragment.

**Table 2:** Conditions of long-range (LR) PCR reactions for *TLR2*, *TLR4*, *TLR9*, *CD14*, *IL23R*, *IL23*, and *IL1R* genes.

Amplified fragment	Polymerase	PCR mixture	PCR conditions
*TLR2* *TLR4* *TLR9* *CD14* *IL23R_1* *IL23R_3* *IL23R_5* *IL23R_6* *IL23* *IL1R*	GoTaq^®^ Long PCR Master Mix (Promega)	57.6 ng of DNA template 0.72 µl of each primer (5 pmol) 15 µl of GoTaq Master Mix Nuclease-free water up to 30 µl	95 °C for 2 min 35 cycles: 94 °C for 30 s 65 °C for 1 min/kbp 72 °C for 10 min Hold at 4 °C
*IL23R_2* *IL23R_4*	FIREPol^®^ DNA Polymerase	80.0 ng of DNA template 1.0 µl of each primer (5 pmol) 0.2 µl of polymerase (500 U) 2.5 µl of buffer 2.0 µl of MgCl_2_ (25 mM) 2.5 µl of dNTPs (5 mM) Nuclease-free water up to 25 µl	95 °C for 4 min 35 cycles: 95 °C for 30 s 58 °C for 30 s 72 °C for 1 min/kbp 72 °C for 7 min Hold at 4 °C

### Library Preparation and Next-Generation Sequencing (NGS)

A total of 35 amplicons from each patient were pooled in equimolar ratios. According to the manufacturer’s protocol, 1 ng of the pooled DNA fragments was subjected to the Nextera XT procedure (Illumina) using transposome technology. Finally, using the Nextera XT DNA Sample Preparation Kit (Illumina) and the Nextera^®^ XT Index Kit (96) (Illumina), we obtained one hundred and seven both-side indexed DNA libraries ready for high-throughput sequencing. The normalization of all libraries was carried out with magnetic beads, according to the Nextera XT procedure. Sequencing on the Illumina MiSeq platform was performed as paired-end targeted resequencing using the MiSeq Reagent Kit v2 (300 cycle) (Illumina). To verify the detected variants, randomly Sanger sequencing analysis was performed on Applied Biosystems 3500 Genetic Analyzer using BigDye^®^ Terminator v3.1 Cycle Sequencing Kit (Thermo Fisher Scientific).

### Bioinformatic Analysis

The generated reads were aligned to the hg19 reference genome with a Burrows–Wheeler Aligner (BWA. version 0.7.5) (Li and Durbin, 2009). PCR duplicates were marked with Picard (version 2.1.0). Local realignment around indels and a base quality score recalibration were performed, followed by naming the variants with the Genome Analysis Toolkit (GATK version 3.5), according to the GATK best practice procedure (McKenna et al., 2010). For detected variants the following quality control (QC) criteria were applied: QualByDepth (QD) < 2.0, FisherStrand (FS) > 60.0, RMSMappingQuality (MQ) < 40.0, MappingQualityRankSumTest (MQRankSum) < -12.5, ReadPosRankSumTest (ReadPosRankSum) < -8.0. Next, single nucleotide polymorphisms/insertions and deletions (SNPs/InDels) were identified by means of using a HaplotypeCaller module. The identified SNPs/InDels were annotated by the VariantAnnotator module. Exons and variants that did not meet our presented NGS quality metrics, were independently analyzed and confirmed with Sanger sequencing. For the haplotype analysis, the Haploview v.4.2 software was used (Purcell et al., 2007).

### Statistical Analysis

The comparison of interval data between responders and nonresponders was conducted by nonparametric Mann–Whitney test, since the data did not follow the normal distribution pattern (Shapiro–Wilk test). The chi-square test was used for comparing nominal data, as well as to determine whether the association between the allele frequencies and the response to treatment was significant (Burke et al., 2018). All *p*-values for detected NGS variants were adjusted by the Benjamini–Hochberg multiple correction. After selecting variants that differed statistically significant in the percentage of particular allele distribution between the group of responders and nonresponders to anti-TNF treatment, in the next step, we used an odds ratio (OR) to demonstrate how many times more often a particular variant occurred in nonresponder patients comparing to responder patients. OR is considered statistically significant when its 95% confidence interval (95% CI) does not contain 1. Moreover, for statistical significant chi-square test results (p<0.05), before using the Benjamini–Hochberg multiple correction, the power calculation test with significance level α=0.05 was performed. The Hardy–Weinberg equation was used to check if the genetic variations of the populations (responders and nonresponders) were at equilibrium. All analyses were performed using STATISTICA 13.3 software (StatSoft, Inc.) and all tests were considered significant at *p* < 0.05.

### Functional Prediction of Identified Variants

For the functional prediction of identified statistically significant variants, an *in silico* analysis of miRNA target sites occurrence in these genes was performed using available databases—miRdSNP and TargetScanHuman 7.2.

## Results

**Figure 1:**
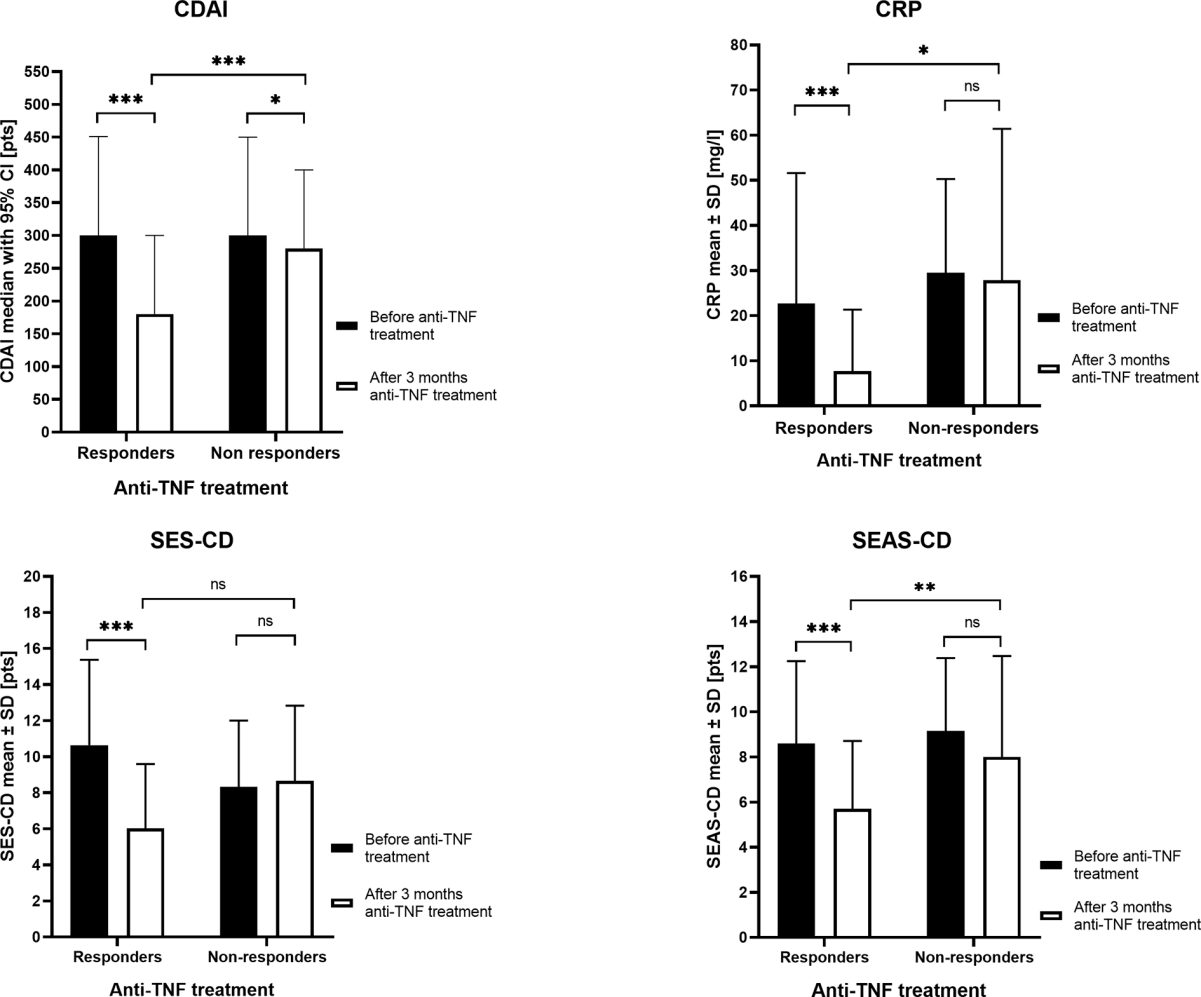
The clinical parameters in responders and nonresponders before anti-tumor necrosis factor (anti-TNF) treatment and after three months of therapy. CDAI, Crohn’s Disease Activity Index; CRP, C Reactive Protein; SES-CD, Simple Endoscopic Score for Crohn’s Disease; SEAS-CD, Simple Enterographic Activity Score for Crohn’s Disease; CI, confidence interval; SD, standard deviation; ns, not significant; *p < 0.05, **p < 0.01, ***p < 0.001.

We studied 107 biologic naive CD patients. Three months after the induction of therapy, 95 patients (88.79%) received a response to the anti-TNF drugs, whereas 12 patients (11.21%) did not respond to treatment. The clinical characteristics of patients are shown in **Table 3** and the most important differences in responders and nonresponders are illustrated in **Figure 1**. In the group of responders, the values of all four parameters used in CD monitoring: CDAI, CRP, SES-CD, and SEAS-CD after induction therapy decreased significantly compared to the state before therapy (*p* < 0.0001). In non-responding patients, parameters remained at a similar level, except for a relatively small decrease in CDAI (*p* < 0.05).

**Table 3:** The characteristics of patients.

Parameter	Results			
	All patients, n = 107	Responders, n = 95	Nonresponders, n = 12	*P*-value
**Gender**, (F/M) n (%)	42 (39.25%)/ 65 (60.75%)	37 (39.95%)/ 58 (61.05%)	7 (58.33%)/ 5 (41.67%)	0.26
**Age**, (years) mean ± SD	28 ± 10.64 [19–64]	29.96 ± 10.36 [28–64]	31.67 ± 13 [19–64]	0.60
**Smoker**, n (%)	8 (7.47%)	8 (9.47%)	0 (0.00%)	0.27
**Previous operations**, n (%)	37 (34.58%)	30 (31.58%)	7 (58.33%)	0.07
**Disease duration**, months, mean ± SD (range)	56.97 ± 48.56 (12–240)	57.85 ± 48.84 (12–240)	50.00 ± 47.48 (12–156)	0.60
**Intestinal location**,* n (%)				
Colonic (L2)	23 (21.50%)	22 (23.16%)	4 (33.30%)	0.44
Ileal (L1)	29 (27.10%)	26 (27.37%)	2 (16.70%)	0.43
Ileocolonic (L3)	55 (51.40%)	47 (49.47%)	6 (50.00%)	0.98
**Behavior**, n (%)				
Nonstricturing, nonpenetrating (B1)	68 (63.55%)	63 (66.32%)	5 (41.67%)	0.10
Stricturing (B2)	4 (3.74%)	3 (3.16%)	1 (8.33%)	0.37
Penetrating (B3)	35 (32.71%)	29 (30.53%)	6 (50.00%)	0.18
Perianal disease modifier (P)	6 (5.61%)	4 (4.21%)	2 (16.67%)	0.08
**Medication**, n (%)				
Mesalamine	106 (99.60%)	95 (100%)	11 (91.67%)	0.01
Corticosteroids	38 (35.51%)	32 (33.68%)	6 (50.00%)	0.26
Azathioprine	67 (63.63%)	59 (62.11%)	8 (66.67%)	0.76
Adalimumab	46 (42.20%)	40 (42.10%)	6 (50.00%)	0.60
Infliximab	61 (57.01%)	55 (57.90%)	6 (50.00%)	0.60

*Disease locations were classiﬁed according to the Montreal Classiﬁcation (Satsangi et al., 2006).

### NGS Analysis

A total of 107 DNA samples obtained from CD patients were analyzed by NGS, using the panel of genes as described above additionally extended with seven genes (**Tables 1** and **2**). They included genes coding for proteins involved in TNF action, expression cascade, signal transmission, and metabolism. According to the clinical procedures (see patients’ characteristics), 12 patients were classified as primary nonresponders to anti-TNF therapy. The NGS analysis results were aligned to the hg19 reference genome and analyzed as previously described (Walczak et al., 2019).

**Figure 2:**
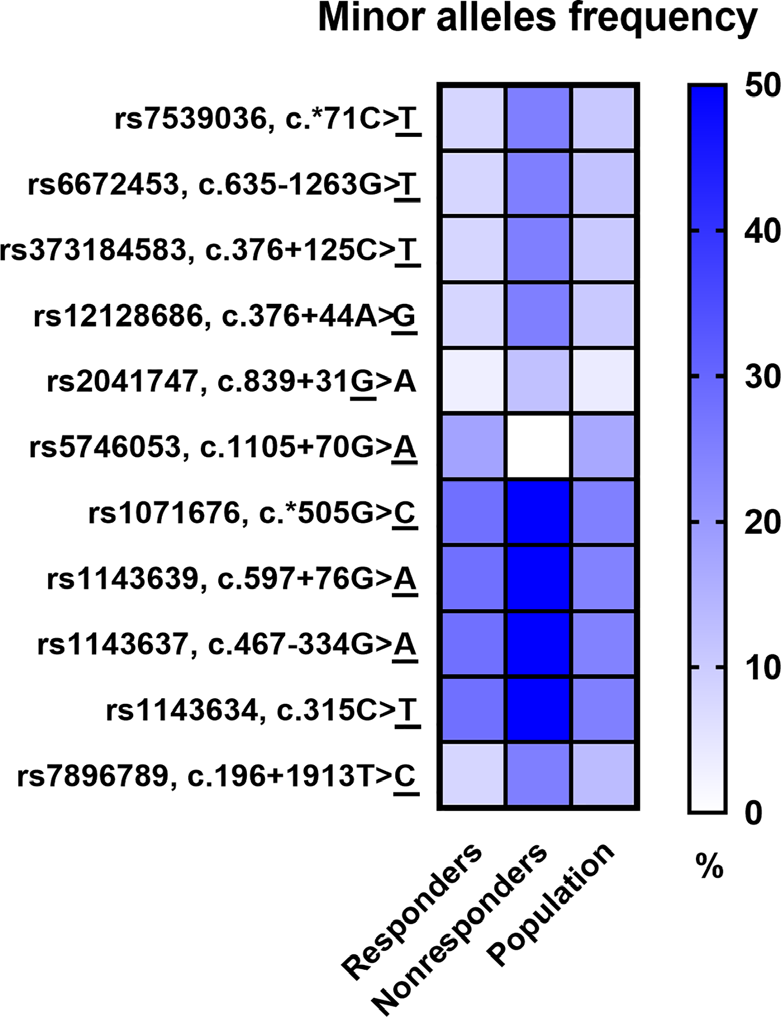
Comparison of minor alleles frequency (MAF) in the study groups and the European population.

The Phred base calling score GQX ≥ 30 of obtained NGS analysis data was an average of 87.45% and passing filter PF 85.91%. After QC analysis and filtering out rare variants with the frequency in the entire study group below 5%, a total of 598 genetic variants were identified, of which 544 (91.0%) were located in introns and 23 (3.6%) in exons. Moreover, 9 (1.5%) changes were observed in the 5’-UTR and 22 (3.7%) in the 3’-UTR. Most of the 598 variants in the number of 540 (90.3%) were SNPs, while the others 58 (9.7%) were InDels. Considering patients’ response to anti-TNF treatment, 11 SNPs revealed statistical significance in a distribution of the genotype frequencies between responders and nonresponders to anti-TNF treatment based on chi-square *p*-value < 0.05. However, after multiple correction the statistical significance was not rich. The majority of these variants were observed in the *FCGR3A* and *IL1B* genes and only single variants in the *TNFRSF1B*, *FAS*, and *IL1R* genes. Results for all *loci* of this group were in Hardy–Weinberg equilibrium (HWE) except for rs5746053 in nonresponders group (**Table 4**). The minor alleles frequencies (MAF) distribution between responders and nonresponders, as well as their comparison with the European population, is shown in **Figure 2**. Nonresponders presented large differences and specified scheme of the alleles distribution for 11 selected *loci* compared to responders and population.

**Table 4:** Genetic variants associated with the response to anti-TNF agents.

No	SNP	Gene	Genotypes							HWE		Alleles		OR	95% CI	*p*-value	Benjamini-Hochberg *p*-value
				Resp			Nonresp			Resp	Nonresp	Resp	Nonresp				
				11	12	22	11	12	22			a2	a2				
1.	rs7539036	*FCGR3A*	n	79	16	0	6	6	0	0.3701	0.2482	16	6	3.63	[1.261–10.425]*	0.01	1.0
			%	83.2	16.8	0	50	50	0			8	25
2.	rs6672453		n	79	16	0	6	6	0	0.3701	0.2482	16	6	3.63	[1.261–10.425]*	0.01	1.0
			%	83.2	16.8	0	50	50	0			8	25
3.	rs373184583		n	79	16	0	6	6	0	0.3701	0.2482	16	6	3.63	[1.261–10.425]*	0.01	1.0
			%	83.2	16.8	0	50	50	0			8	25
4.	rs12128686		n	79	16	0	6	6	0	0.3701	0.2482	16	6	3.63	[1.261–10.425]*	0.01	1.0
			%	83.2	16.8	0	50	50	0			8	25
5.	rs2041747	*IL1R*	n	90	5	0	9	3	0	0.7922	0.6207	5	3	5.29	[1.178–23.708]*	0.02	1.0
			%	94.7	5.3	0	75	25	0			3	12
6.	rs5746053	*TNFRSF1B*	n	65	26	4	12	0	0	<0.0001	0.5036	34	0	0.09	[0.005–1.560]	0.02	1.0
			%	68.4	27.4	4.2	100	0	0			18	0
7.	rs1071676	*IL1B*	n	52	33	10	2	8	2	0.1834	0.2482	53	12	2.59	[1.093–6.113]*	0.03	1.0
			%	54.8	34.7	10.5	16.7	66.8	16.7			28	50
8.	rs1143639		n	52	33	10	2	8	2	0.1834	0.2482	53	12	2.59	[1.093–6.113]*	0.03	1.0
			%	54.8	34.7	10.5	16.7	66.8	16.7			50	50
9.	rs1143637		n	52	33	10	2	8	2	0.1834	0.2482	53	12	2.59	[1.093–6.113]*	0.03	1.0
			%	54.8	34.7	10.5	16.7	66.8	16.7			28	50
10.	rs1143634		n	52	33	10	2	8	2	0.1834	0.2482	53	12	2.59	[1.093–6.113]*	0.03	1.0
			%	54.8	34.7	10.5	16.7	66.8	16.7			28	50
11.	rs7896789	*FAS*	n	80	14	1	8	2	2	0.7839	0.0543	16	6	3.63	[1.261–10.425]*	0.03	1.0
			%	84.2	14.7	1.1	66.8	16.7	16.7			8	25

SNP, single nucleotide polymorphisms; Resp, responders; Nonresp, nonresponders; HWE, Hardy-Weinberg equilibrium; *95% CI confirming the statistically significant odds ratio (OR); 11 – the first type of homozygous genotype; 22 – the second type of homozygous genotype; 12 – genotype heterozygote; a1 – the first allele; a2 – the second allele.

### Haplotype Analysis

**Figure 3:**
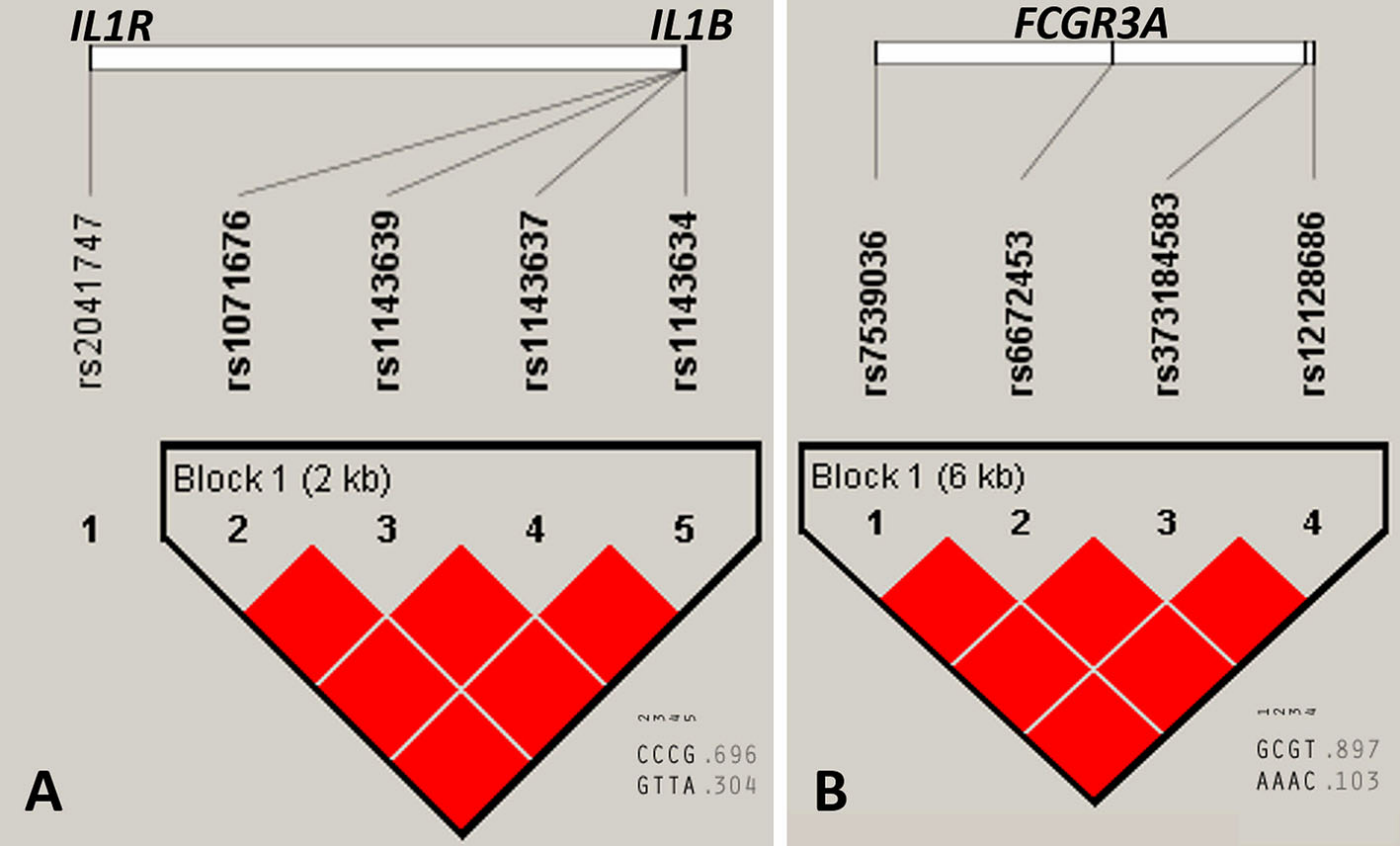
Linkage disequilibrium (LD) plots of selected gene variants for the whole study group. **(A)** *IL1R* and *IL1B* genes, **(B)** *FCGR3A* gene. Numerical values below (0.696, 0.304, 0.897, and 0.103) represent haplotype frequencies. Red color of squares indicates D’ = 1, where the D’ represent statistics for each pairwise comparison. The LD plot was generated with Haploview v.4.2 software (Gabriel et al., 2002; Barrett et al., 2005).

Linkage disequilibrium (LD), determined in Haploview v.4.2, for selected above described variants, which were located in neighborhood on the same chromosome, showed that *loci* within *IL1B* and *FCGR3A* genes constructed two haploblocks in complete LD (D’ = 1) for the whole study group (**Figure 3**). The value of r^2^ was 1 for all pairwise comparisons within both genes. The nucleotide substitutions in the *IL1R* gene (rs2041747) was not significantly linked with *IL1B* gene variants located also on the longer arm of chromosome 2 (**Figure 3**).

### Functional Prediction of Identified Variants

The identified variants were predominantly intronic. However, the occurrence of genetic variants in exonic, and 3’-UTR regions were observed (**Table 5**). The exonic variants are important due to the potential impact on the gene expression profile. The 3’-UTR SNP polymorphisms were identified in *IL1B* and *FCGR3A* genes. The *in silico* analysis identified that rs1071676 in the *IL1B* gene is located on the target site for has-miR-622, while rs7539036 in the *FCGR3A* gene is located on the target site for hsa-miR-6890-5p, hsa-miR-193b-5p, hsa-miR-3170, and hsa-miR-6855-5p (**Table 5**). This suggests a possible role of these polymorphisms in the control of the mRNA expression level.

**Table 5:** Predicted functional effect of the identified variants.

No.	Gene	SNP	DNA	Protein	Variant	Functional effect *in silico*	Reference
1.	*FCGR3A*	rs373184583	c.376+125C>T	–	Intron	Unknown	–
2.	*FCGR3A*	rs12128686	c.376+44A>G	–	Intron	Unknown	–
3.	*FCGR3A*	rs7539036	c.*71C>T	–	3’-UTR	hsa-miR-6890-5p hsa-miR-193b-5p hsa-miR-3170 hsa-miR-6855-5p	TargetScanHuman 7.2
4.	*FCGR3A*	rs6672453	c.635-1263G>T	–	Intron	Unknown	–
5.	*IL1R*	rs2041747	c.839+31G>A	–	Intron	Unknown	–
6.	*TNFRSF1B*	rs5746053	c.1105+70G>A	–	Intron	Unknown	–
7.	*IL1B*	rs1071676	c.*505G>C	–	3’-UTR	hsa-miR-622	TargetScanHuman 7.2
8.		rs1143639	c.597+76G>A	–	Intron	Unknown	–
9.		rs1143637	c.467-334G>A	–	Intron	Unknown	–
10.		rs1143634	c.315C>T	p.F105=	Exon	Synonymous	dbSNP
11.	*FAS*	rs7896789	c.196+1913T>C	–	Intron	Unknown	–

UTR, untranslated region.

## Discussion

In recent years, numerous studies concerning IBD pharmacogenetics have highlighted the need to better understand the mechanism of response to biological treatment, as well as to develop personalized therapeutic strategies (Parsi et al., 2002; Horiuchi et al., 2010; Trent, 2010; Bek et al., 2016; Prieto-Perez et al., 2016; De Lange et al., 2017). Up to now, many genes have been investigated, particularly the members of the TNF family. However, the results are often contradictory and are still far from defining the biomarkers of the anti-TNF response (Leal et al., 2015a; Medrano et al., 2015; Ternant et al., 2015). Additionally, there are no studies on the Polish population.

NGS technology has certainly revolutionized genomic research. However, despite its high capacity, it seems to be relatively expensive for sequencing genes that do not already occur in the commercially available panels (Li and Durbin, 2009). Therefore, by using NGS combined with LR-PCR libraries, we attempted to determine genetic predictors and share our conclusions of investigating a cohort of 107 Polish IBD patients treated with anti-TNF agents who presented various responses to the therapy. Our molecular genetic results of 23 selected genes indicated that variants of only five genes (*TNFRSF1B*, *FCGR3A*, *FAS*, *IL1R*, and *IL1B*) presented a significant correlation with the response. The related genes encoded mostly receptor proteins (FcgRIII, TNF-R2, IL-1R-1, and FasR) and additionally cytokine IL-1 beta involved in the apoptotic process. In fact, the genetic variability in receptors could potentially account for the nonresponse phenomenon, since one possible mechanism of anti-TNF nonresponse is T cells’ insensitivity to apoptosis signals.

We observed the majority of significant variants in the *FCGR3A* gene, which codes for a receptor of the Fc part of immunoglobulin G and plays a key role in the mechanism described as antibody-dependent cell cytotoxicity (ADCC). In our CD patient cohort, for four genetic variants (rs7539036, rs6672453, rs373184583 and rs12128686) the minor allele (T, T, T and G, respectively) was almost four times more common in the nonresponders group (OR: 3.63, 95% CI: 1.261–10.425; **Table 4**), which may indicate that it is associated with a nonresponse to biological treatment. Moreover, rs373184583, rs12128686, and rs667245 are located in introns, whereas rs7539036 (c.*71C>T) was located in the 3′ UTR. *In silico* analysis identified the UTR location of the rs7539036 variant as occurring in miRNA’s binding region (**Table 5**). However, its potential functional effect and translation into clinical consequences have to be validated experimentally. In fact, the genetic amino acid substitution p.V158F (rs396991) in the *FCGR3A* gene, which is well described in the literature, was found to affect the response to antibodies treatment in the case of inflammatory bowel disease (Louis et al., 2004; Moroi et al., 2013; Louis et al., 2016; Romero-Cara et al., 2018). However, the association between this polymorphism and the clinical response in CD has also been questioned (Slebioda and Kmiec, 2014), and it is consistent with our results.

The tumor necrosis factor receptor superfamily member 1B is one of two TNF receptors. Its expression can be mostly found on lymphocytes, epithelial cells, and subtypes of neurons (Matsukura et al., 2008; Horiuchi et al., 2010). Therefore, it is an obvious target in pharmacogenetic investigations. In this study, one SNP rs5746053 in the *TNFRSF1B* gene was identified as possibly associated with a response to biological treatment. The minor allele A occurred significantly frequently in the group of responders (18%), as compared to the nonresponders (0%), which may indicate the minor allele’s protective effect. This SNP may affect the gene expression level, for example, by changing the sequence of the transcription factor binding sites, however that remain unknown and the further research are needed. In the *TNFRSF1B* gene, rs1061624 was previously identified as related to the reaction in a haplotype with rs3397 in the Japanese population in AT configuration, respectively (Medrano et al., 2014). An increased frequency of rs1061624_A-rs3397_T haplotype in patients classified as nonresponders was also confirmed by studies performed on a Spanish cohort of CD patients (Strasser et al., 2009), however, in our research, we have not observed this association.

Another association with the anti-TNF response was found for the Fas cell surface death receptor (FAS), a member of the tumor necrosis factor receptor family containing a death domain and playing a central role in programmed cell death. Activated by the FAS ligand (FASL), it triggers apoptosis. FASL expressed on activated T and NK cells contribute to their ability to kill targets (Trambas and Griffiths, 2003; Leal et al., 2015b). One intronic polymorphism was identified as possibly associated with a lower response in this study, namely rs7896789. This genetic variant has been described for the first time; however, its clinical implications remain unknown.

IL-1β transduces the signals of the inflammatory response and activates other cytokines by binding to the IL-1 receptor (IL-1R). An elevated level of IL-1β has been related to a number of diseases. Studies have shown that IL-1β is upregulated in refractory to anti-TNF treatment CD patients (Leal et al., 2015b) and ulcerative colitis patients (Dahlén et al., 2015). Lacruz-Guzman and colleagues described the potential pharmacogenetic role of rs1143634 (c.315C>T). The C allele occurrence was associated with a lower response to infliximab treatment in CD patients, as well as with the higher serum concentration of IL-1β (Lacruz-Guzmán et al., 2013). In our group of Polish patients, the SNP was also identified as related to an anti-TNF response, however, the minor allele T was more frequent in nonresponders (50% *vs* 28%, respectively). The rs1143634 variant occurs in an exonic part of the gene. However, it is synonymous and does not cause amino acid variant. Additionally, we described rs1071676 (c.*505G>C) for the first time as a potential pharmacogene in CD. This variant occurs in the 3’-UTR region. By *in silico* analysis, we identified it as a variant which could affect miRNA activity (**Table 5**), and its potential functional effect is being investigated. For the remaining two variants of the *IL1B* gene [rs1143639 (c.597+76G>A) and rs1143637 (c.467-334G>A)], in terms of the intronic variant found in the interleukin 1 receptor gene (*IL1R*) [rs2041747 (c.839+31G>A)], the relationship with an anti-TNF response is described for the first time in this paper—their functional effect has not been previously studied.

Recently, Danish researchers analyzed 53 biologically functional SNPs involved in the NFκB, the TNF, and other cytokine pathways and showed that 10 variants in the NFκB pathway [*TLR2* (rs11938228), *TLR4* (rs5030728 and rs1554973), and *NFKBIA* (rs696)], in the TNF-α pathway [*TNFRSF1A* (rs4149570)], and in other proinflammatory pathways [*IL1RN* (rs4251961), *IL18* (rs1946518 and rs187238), *NLRP3* (rs4612666), and *JAK2* (rs12343867)], were associated with a response to anti-TNF therapy on a large cohort of patients (587 CD and 458 ulcerative colitis (UC)) (Bank et al., 2019). For *TNF*, *TNFRSF1A*, *TLR2*, and *TLR4* genes, our results do not confirm the observations of Bank and coauthors. Other genes were not included in our panel; however, it would be beneficial to extend our long-range PCR libraries and NGS methodology with the *NFKBIA*, *IL1RN*, *IL18*, *NLRP3*, and *JAK2* genes and to verify the data for the Danish cohort in our CD patient group.

**Figure 4:**
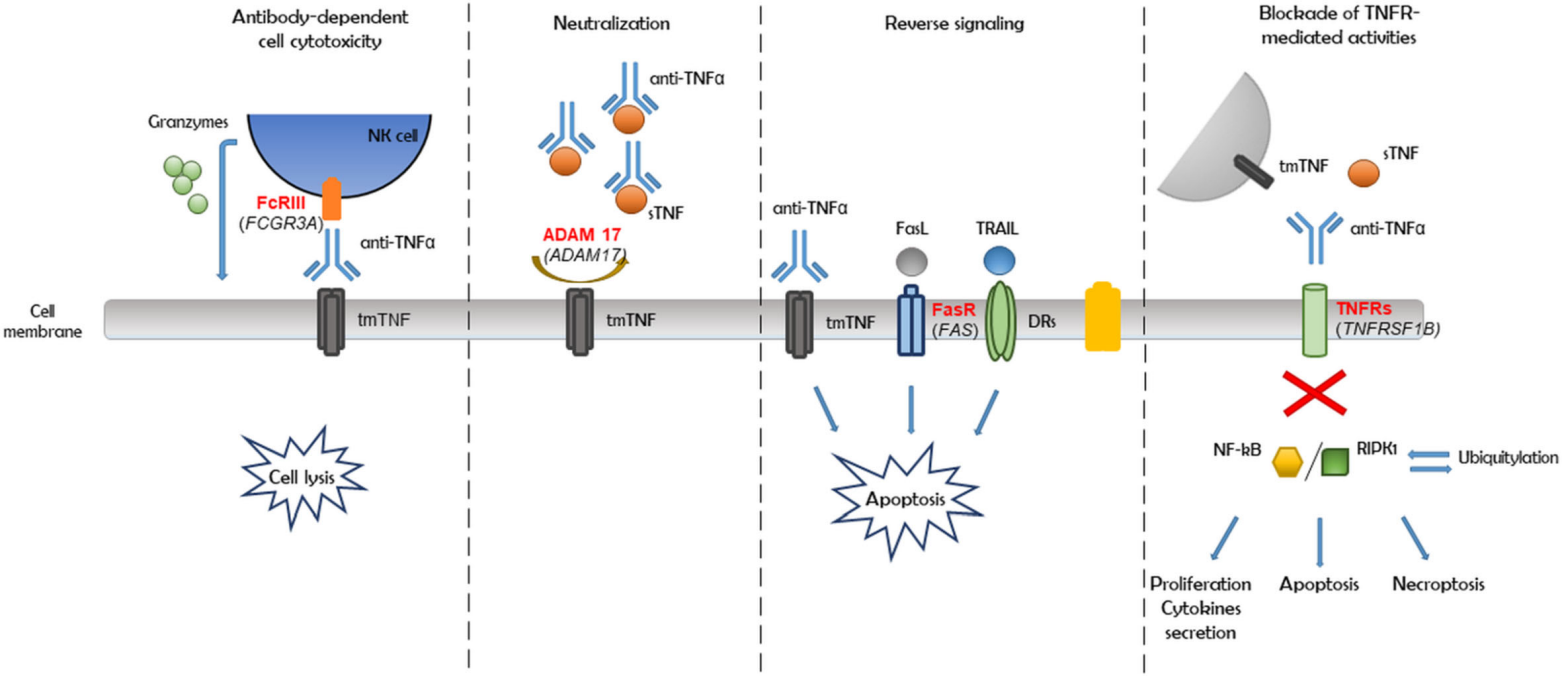
Pathways of anti-TNF monoclonal antibodies action in Crohn's disease (CD).

Furthermore, based on haplotype analysis, we observed that most of the detected variants were linked within genes (**Figure 3**). Therefore, all 11 genetic variants associated with a nonresponse may not be substantially involved in a patient’s reaction to the anti-TNF therapy. Moreover, an association could only be a consequence of linkage and a close location in the chromosome. On the basis of the abovementioned observations, the potential mechanism of nonresponse could be driven by independent inflammatory or apoptosis pathways, such as antibody-dependent cell cytotoxicity, reverse signaling, and the blockade of TNF receptor-mediated activities, as well as the neutralization of the anti-TNF drug due to impaired convertase ADAM 17, which together would eventually cause an adverse effect (**Figure 4**).

### Study Limitations and Strengths

There are several limitations of our study. The first is a lack of measurement of drug levels and antidrug antibodies. However, the vast majority of data in this respect is related to the phenomenon of a secondary loss of response during long-term maintenance treatment. Two strategies have been proposed in terms of therapeutic drug monitoring (TDM) in IBD—proactive and reactive. However, none of them has been verified in patients undergoing the induction treatment. It seems that in the case of primary nonresponse other factors (such as genetics) could play a significantly more substantial role. The second limitation could be the size of our study group. We conducted the sequencing analysis of whole genes including numerous variants (nearly 600 as seen from the results) and not only single selected genetic variants. The power calculation performed after chi-square test for 11 associated variants (p<0.05), before using the Benjamini–Hochberg multiple correction, ranged from 0.44 to 0.82. In medical studies the desired power is more than 0.80. This means that our study could be underpowered in some proportion of many genetic variants analyzed in the current study. This is an obvious consequence of the study group size, however we included all available patients qualified for biological treatment in 2013–2017 in our center (which, in turn, is independently regulated by the reimbursement rules of the Polish National Health System). Another possible limitation is that we assessed the DNA variants in the peripheral lymphocytes and not the genes expression directly in the inflamed tissue obtained endoscopically. Nevertheless, these results were essential to plan further, functional genetic analyses and our study also has a few strengths. The investigated group was ethnically homogenous and the participants had similar CD severity. In this study, we performed for the first time a complex analysis regarding the genetic background of the nonresponse phenomenon in CD patients treated with anti-TNFα drugs. In the majority of cases, the described SNPs are located in intronic regions, 3’-UTR and only one is located in exonic sequence. Special attention must now be paid to variants in the UTR regions, whose role has been underestimated due to the focus on the gene coding regions. Recently, noncoding regions have also been widely studied in the literature, and their functional importance at the protein level has been confirmed (Anbazhagan et al., 2019; Durmanova et al., 2019). In our study, the described variants could lead to changing protein activity, eventually affecting the mechanism of the antibodies action and explain the phenomenon of refractory patients. Exploring their participation at the protein level requires functional testing. These variants could potentially affect RNA splicing by altering splice sites, branch points, or intronic enhancer/silencer motifs (Mahaweni et al., 2018).

In fact, functional analysis results are crucial to confirm the importance of detected variants. However, these results may constitute a basis for further studies, which can be a step toward the detection of predictive biomarkers in anti-TNF treatment. In the biological treatment era, they are strongly desirable and needed in clinical practice, as well as in personalized treatment. We currently have more and more new biological molecules at our disposal. This may allow for a better choice of biological drugs for individual patients, hence, reducing the risk of adverse drug reactions and strong interference in the immune system—the long-term consequences of which are hard to anticipate today.

## Data Availability Statement

The raw data supporting the conclusions of this article will be made available by the authors, without undue reservation.

## Ethics Statement

The studies involving human participants were reviewed and approved by Bioethical Committee of the University of Medical Sciences in Poznan, Poland, under Resolution No. 762/13. The patients/participants provided their written informed consent to participate in this study.

## Author Contributions

Conceptualization. LL-S and MS-Z. Methodology and validation, MW, LL-S, and OZ-B. Investigation, WMW, LL-S, OZ-B, IK-K, PE, KS-E, and MS. Formal analysis, MP, MZ, MM, and WMK. Writing—original draft preparation, MW, LL-S, and MS-Z. Critical revision of the manuscript, PE and AD. Funding acquisition, KS-E and RS. Supervision, AD, RS and MS-Z. Acceptance of the final version, all authors.

## Funding

This work was supported by the Foundation for the Development of Biotechnology and Genetics (POLBIOGEN). KS-E was the recipient of a fellowship for young researchers from Poznan Medical University, Poland (grant no. 502–14–02223359–10715).

## Conflict of Interest

The authors declare that the research was conducted in the absence of any commercial or financial relationships that could be construed as a potential conflict of interest.
